# Comparative analyses on the structure, composition, and gene expression in seed coat of hulled and hull-less lines in *Cucurbita moschata*

**DOI:** 10.1186/s12870-026-09558-8

**Published:** 2026-07-24

**Authors:** Qiong Shen, Jiangtao Liu, Hongyu Meng, Yanan Liu, Jianxin Shi

**Affiliations:** 1 College of Horticulture, Shanxi Agricultural University, Taiyuan, 030031 China; 2 Joint International Research Laboratory of Metabolic and Developmental Sciences, School of Life Sciences and Biotechnology, Shanghai Jiao Tong University, Shanghai, 200240 China; 3 Hainan Research Institute, Shanghai Jiao Tong University, Sanya, 572024 China

**Keywords:** *Cucurbita moschata*, Hull-less, Seed coat, Starch, Lignin, Cellulose, Hemicellulose, Pectin

## Abstract

The escalating demand for hull-less *Cucurbita moschata* seeds, driven by a thorough comprehensive understanding of their functional properties and the global trade in pumpkin seeds, emphasizes the necessity to expedite the breeding of hull-less varieties to satisfy market requirements. Existing knowledge regarding hull-less seeds in pumpkins primarily stems from studies on *C. pepo*, where the lignin pathway is considered a significant factor; however, its significance in *C. moschata* remains unexplored. This study aims to investigate anatomical, compositional, and gene expression alterations in the seed coat of one hulled wild type (RHS-1) and three hull-less mutant recombinant inbred lines (RHLS-2, RHLS-3 and RHLS-4) at 20 DPA (days post anthesis) and 50 DPA in *C. moschata*. The anatomical changes in the seed coat of hulled and hull-less lines in *C. moschata* during seed coat development were comparable to those observed in *C. pepo*, with five distinct seed coat layers appearing in the hulled wild type but a papery thin layer (composed of the epidermis and sclerenchyma) in the hull-less mutants. However, compositional changes in pectin content in the seed coat of hulled and hull-less lines in *C. moschata* differed from those in *C. pepo*. Specifically, the seed coat of hull-less mutants exhibited higher pectin levels than that of hulled wild type. Additionally, the expression of three lignin biosynthesis genes (*CmoC4H, Cmo4CL*, and *CmoCCR*) was upregulated at 50 DPA, which was contrary to the patterns observed in *C. pepo*. In summary, our results revealed both conserved and distinct features of the seed coat development between *C. moschata* and *C. pepo*. These correlative findings provide comparative features of seed coat development in hulled and hull-less *C. moschata* lines and provide candidate genes for future functional studies.

## Introduction

Pumpkin seeds are rich in unsaturated fats, proteins and carbohydrates, which positions them as a potential functional food [1, 2]. Pumpkin seed oil also contains health-related phytochemical such as carotenoids, squalenes and tocopherols. Consequently, it holds promise for addressing cardiovascular problems and diseases associated with hormonal imbalance in menopausal women [3–5]. However, the laborious process of removing the seed coat poses limitations on the development of both seed and oil industry in pumpkin. The hull-less seed trait presents a genetic characteristic that eliminates this tedious task while enhancing oil production efficiency [6–8]. Thus, the manipulation of seed coat formation holds considerable potential for diverse commercial applications of pumpkin seeds.

The two primary cultivated species of *Cucurbita* are *Cucurbita moschata* and *Cucurbita pepo,* both of which have seen rapid advancements in fruit production and a substantial scale in seed production. In China, pumpkin seed production accounts for about 70% of the global market, with an estimated 290,000 hectare in 2017. Consequently, the pumpkin seed production has become a major sector of for pumpkin production in China [9, 10]. Nevertheless, compared to *C. pepo,* knowledge about the physiological, biochemical, and molecular aspects of hull-less seeds are fragmented [6–8]. A systematic identification and characterization of hull-less mutants will pave the way for broader application of pumpkin seeds, benefiting both farms and relevant industries.

The first spontaneous hull-less mutant in pumpkin was reported in *C. pepo* around 1880 s [11]. The mutant is controlled by a single recessive gene *h* (for *hull-less seed*) [12]. Anatomical studies have revealed that the seed coat of wildtype seeds in *C. pepo* consists of typical five cell layers, epidermal (E), hypodermis (H), sclerenchyma (S), aerenchyma (A), and chlorenchyma (C), whereas in hull-less mutants, the collapse of four seed coat tissue layers (E, H, S and A) results in a papery thin seed coat [12–15]. Physiological and biochemical analyses revealed that lignin and cellulose contents, as well as the activities of key lignin biosynthetic enzymes, such as phenylalanine ammonia lyase (PAL), 4-coumaric acid coenzyme A ligase (4CL), cinnamate-4-hydroxylase (C4H), cinnamyl alcohol dehydrogenase (CAD), and peroxidase (POD), are significantly reduced in hull-less mutants after approximately 20 days post-anthesis (DPA) [16–19]. Gene expression analyses further demonstrated that genes encoding these enzymes show differential expression patterns between hulled and hull-less genotypes [20]. Recent genetic studies have fine-mapped the hull-less locus to chromosome 12 in *C. pepo*, identifying *CpNST1*, a gene encoding a NAC transcription factor involved in secondary cell wall biosynthesis, as the causal gene for the hull-less trait [6, 21–23].

Beyond *Cucurbita*, studies in other plant species have provided valuable insights into seed coat development and lignification. In *Arabidopsis thaliana*, the seed coat consists of five distinct layers, with the inner integument undergoing lignification during development, and transcription factors such as *NST1* and *MYB46* have been identified as master regulators of secondary cell wall formation through the phosphorylation-mediated regulation of *NST1* activity [24] and *MYB46* protein stability [25]. In rice, NAC transcription factors have also been shown to directly regulate lignin biosynthesis. For example, *OsNAC055* activates the expression of key lignin biosynthetic genes such as *OsCCR10* and *OsCAD2* [26], and *OsNAC1* positively modulates the expression of multiple lignin biosynthetic genes to promote lignin accumulation [27]. In oilseed rape (*Brassica napus*), multi-omics analyses have revealed that genes such as *CCRL* and *TT8* modulate lignin biosynthesis and seed coat content, and comparative transcriptomic analysis has demonstrated that high-lignin lines exhibit significantly thicker seed coats with upregulation of *BnPAL*, *BnC4H*, *Bn4CL*, *BnNAC080*, and *BnMYB9* [28, 29]. These findings across diverse species suggest a conserved regulatory module involving NAC transcription factors and downstream lignin/cellulose biosynthetic genes in seed coat development. Whether it is applicable to *Cucurbita* remains unknown.

Hull-less seeds of *C. moschata* are considered superior in taste compared with those of *C. pepo*, making them highly desirable for culinary use [30]. The hull-less *C. moschata* mutant was first discovered by our laboratory in the 1980 s, with its inheritance controlled by a pair of alleles: "NN" (hulled) and "nn" (hull-less) [31, 32]. Anatomical studies have revealed that the seed coat of hulled *C. moschata* exhibits a typical five-cell layer structure comparable to that of *C. pepo*. During seed coat development, lignification gradually occurs, particularly in the S layers, persisting in mature seeds of hulled lines but lacking in that of hull-less seeds [7, 8]. Physiological analyses further demonstrated that reduced lignin content leads to seed coat collapse in hull-less mutants, accompanied by significantly reduced activities of lignin biosynthetic enzymes (PAL, 4CL, CAD, and C4H) after 20 DPA, with 4CL activity declining from 15 DPA [7]. Using bulked segregant analysis sequencing (BSA-seq), a single recessive gene (N) was mapped to a 2.4 Mb region on Chromosome 17, and *CmoCh17G004790*, encoding the NAC transcription factor *CmNST1*, was identified as the most probable candidate gene. Transcriptomic profiling further confirmed *CmNST1* as a master regulator of lignin biosynthesis during seed coat development, with other NAC and MYB transcription factors also contributing to the regulatory network for secondary cell wall formation [8]. Nevertheless, alterations in the structure, composition, and gene expression in the seed coat of hull-less seeds in *C. moschata* remain uninvestigated.

In this study, we conducted a comprehensive comparative analysis of the anatomical, compositional, and gene expression characteristics of three hull-less mutants relative to the hulled wild type (WT). Our objectives were (1) to investigate the anatomical variations among different hull-less mutants; (2) to elucidate the underlying changes in seed coat composition, and gene expression, as well as their correlations with seed coat development. The relevant data presented in this study contribute to our understanding of *C. moschata* seed coat development and provide a preliminary basis for exploring its industrial applications.

## Materials and methods

### Materials

Parent inbred lines of *C. moschata* with hulled seed (HS-A) and hull-less seed (HLS-B) were obtained from the College of Horticulture, Shanxi Agricultural University (SAU), Taiyuan, China. A recombinant inbred line (RIL) population with 189 F_8_ RILs was developed from the cross between the two inbred lines through single seed descent (SSD). Based on the criterion of whether seeds can be consumed directly without dehulling, RHS-1 was regarded as the hulled wild type line (consumed after dehulling), while RHLS-2, RHLS-3, and RHLS-4 as three hull-less mutant lines (consumed directly without dehulling). RHLS-2, RHLS-3, and RHLS-4 exhibit moderate, extensive, and nearly complete seed coat degradation phenotype, respectively. Ten plants of each line were cultivated in plastic greenhouses at the Dongyang Innovation Base of Shanxi Agricultural University (SAU) in Jinzhong, Shanxi Province. Plants were grown at a row spacing of 1.0 m and a plant spacing of 0.5 m, with vines suspended vertically, following standard local agronomic practices for *C. moschata* cultivation. Self-pollination was performed at anthesis to ensure fruit set. Seeds and seed coats were collected at 20 and 50 days post anthesis (DPA). For electronic microscopic observations, 30 seeds per line were randomly sampled at each stage (10 each from each young fruit, three young fruits from each line) were sampled. For biochemical analysis of seed coat composition and qRT-PCR analysis, seed coats of each fruit were pooled (10 seeds of each young fruit) and three young fruits of each line were taken as three biological replicates. The collected samples were stored in −80℃ until further analysis. The developmental stages of 20 DPA and 50 DPA were selected based on previous physiological characterizations of seed coat development in *C. moschata* (Shen et al., 2019, 2023). 20 DPA represents a critical morphogenetic stage during which lignin biosynthesis enzyme activities begin to diverge between hulled and hull-less lines, showing no visible distinct whole-seed phenotypes, while 50 DPA corresponds to physiological maturity, showing clear visible distinct whole-seed phenotypes between hulled and hull-less lines.

### Phenotypic observation

The pre-treated samples were rinsed with 0.1 M phosphate buffered salt solution (PBS), dehydrated by gradient ethanol treatment series (30%, 50%, 70%, 80%, 95%, and 100% each for 15 min), and dried in a supercritical carbon dioxide device. Dried samples were placed in order on the stage, sprayed with gold, and observed under a scanning electron microscope (SEM, JEOL JSM-6701F) at 10 kV. The images were analyzed using Leica Cytogenetics Software (Laboratory Imaging Software, Prague, Czech Republic).

### Biochemical composition analysis

Biochemical composition of seed coat was measured using corresponding assay kits from Solarbio Life Sciences (Beijing, China) following the manufacturer’s protocols. Specifically, the content of starch, total pectin, hemicellulose, cellulose and lignin of seed coat was measured using the Starch Content Assay Kit (Cat#BC0705), the Total Pectin Content Assay Kit (Cat#BC1405), the Hemicellulose Content Assay Kit (Cat#BC4440), the Cellulose (CLL) Assay Kit (Cat#BC4280), and the Lignin content Assay kit (Cat#BC4200), respectively.

Absorbance values for lignin were measured at 280 nm using a spectrophotometer. Cellulose, starch, hemicellulose, and pectin were measured using an ELISA reader at 620 nm, 620 nm, 540 nm, and 530 nm, respectively. Standard curves were constructed using respective standard substances, and the content of each component was calculated according to the following formulas.

$\mathrm{L}\mathrm{i}\mathrm{g}\mathrm{n}\mathrm{i}\mathrm{n} \mathrm{c}\mathrm{o}\mathrm{n}\mathrm{t}\mathrm{e}\mathrm{n}\mathrm{t} \left(\mathrm{m}\mathrm{g}/g\right)=2.184\times \Delta \mathrm{A}/W$, where ΔA is the change in absorbance, and W is the dry sample weight (0.005 g).

$\mathrm{C}\mathrm{e}\mathrm{l}\mathrm{l}\mathrm{u}\mathrm{l}\mathrm{o}\mathrm{s}\mathrm{e} \mathrm{c}\mathrm{o}\mathrm{n}\mathrm{t}\mathrm{e}\mathrm{n}\mathrm{t} \left(mg/g\right)=22.52\times x\times {W}_{2}/{W}_{3}/{W}_{1}$, where x is the concentration derived from the standard curve, W_1_ is the sample weight (0.3 g), W_2_ is the cell wall material (CWM), and W_3_ is the dried CWM weight (0.005 g).

$\mathrm{H}\mathrm{e}\mathrm{m}\mathrm{i}\mathrm{c}\mathrm{e}\mathrm{l}\mathrm{l}\mathrm{u}\mathrm{l}\mathrm{o}\mathrm{s}\mathrm{e} \mathrm{c}\mathrm{o}\mathrm{n}\mathrm{t}\mathrm{e}\mathrm{n}\mathrm{t} \left(mg/g\right)=y/W\times F$, where y is the concentration derived from the standard curve, W is the sample weight (0.05 g), and F is the dilution factor.

$\mathrm{S}\mathrm{t}\mathrm{a}\mathrm{r}\mathrm{c}\mathrm{h} \mathrm{c}\mathrm{o}\mathrm{n}\mathrm{t}\mathrm{e}\mathrm{n}\mathrm{t} \left(mg/g\right)=0.811\times x/W\times F$, where x is the concentration derived from the standard curve, W is the sample weight (0.03 g), and F is the dilution factor. The constant 1.11 was used to convert glucose content to starch content.

$\mathrm{P}\mathrm{e}\mathrm{c}\mathrm{t}\mathrm{i}\mathrm{n} \mathrm{c}\mathrm{o}\mathrm{n}\mathrm{t}\mathrm{e}\mathrm{n}\mathrm{t} \left(\upmu \mathrm{m}\mathrm{o}\mathrm{l}/g\right)=x/W$, where x is the absorbance value and W is the sample weight (0.05 g).

All measurements were performed in three biological replicates.

### RNA extraction and qRT-PCR analysis

Total RNA was extracted from the seed coat samples using RNAprep Pure Plant Kit (Tiangen Co., Beijing, China). cDNA was synthesized using ReverTra Ace qPCR RT Master Mix with gDNA Remover (Code No. FSQ-301, TOYOBO, JAPAN). Quantitative real-time PCR (qRT-PCR) was performed using TB Green® Premix Ex Taq™ II. qRT-PCR was performed with 1 μL of cDNA, 0.8 μL of each upstream or downstream primer, 10 μL of TB Green Premix Ex Taq and 7.4 μL of ddH_2_O (20 μL total) under the following conditions: initial denaturation at 96 ℃ for 30 s, followed by 45 cycles of denaturation at 95 ℃ for 15 s, annealing at 60 ℃ for 30 s, and final extension at 72 ℃ for 30 s. Three technical replicates were used for each biological replicate, three biological replicates were used for each line. Based on prior evidence, nine structural genes were selected for expression analysis, including five lignin-related genes (*CmoPAL*/*CmoCh07G009550*, *CmoCAD*/*CmoCh01G020440*, *CmoC4H*/*CmoCh02G006920*, *Cmo4CL*/*CmoCh01G005470*, and *CmoCCR*/*CmoCh17G008650*) as orthologs of previously characterized lignin biosynthetic genes in *C. pepo* [20], and four cellulose synthase genes (*CmoCh03G008270*, *CmoCh10G004910*, *CmoCh11G004480*, and *CmoCh17G013330*) selected as the most highly expressed *CesA* genes during seed coat development from our transcriptomic data [8]. Primers were designed by Primer Premier 3.0 software and the *Actin* gene was used as the internal reference (Table 1). Relative expression levels were quantified using the 2^−ΔΔCT^ method [33], and significance analysis was performed using DPS 9.50 software.

**Table 1:** The sequences of primers used in this study

Gene No	Primer sequences (5’−3’)	Length (bp)
*CmoPAL* (*CmoCh07G009550*)	F:GCGCATGGTGGAGGAATATC	114
	R:GCCAACTCGACCATCACATC	
*CmoCAD* (*CmoCh01G020440*)	F:CGAATCACACACAGACGGTC	108
	R:CTATCGTCACGTCGTCTGGA	
*CmoC4H* (*CmoCh02G006920*)	F:ATGGTGAGCACTGGAGGAAA	94
	R:TCGTACTCCCATCCGTAACG	
*Cmo4CL* (*CmoCh01G005470*)	F:TCTGGCTCCGAATTCCCAAT	82
	R:TGGCTCCTGTTGTTCCTGAT	
*CmoCCR* (*CmoCh17G008650*)	F:AGGTTGCGAAGGAGAAGGAA	86
	R:GGCATTAATGGTGGGTTGCA	
*CmoCh03G008270*	F:AAGGGAGATGCTGCTGAGTT	143
	R:GCACGGACAACTGATAGCTG	
*CmoCh10G004910*	F:GATGGAAGAAGGCGGTGTTC	130
	R:AGAACCGTAAATCCAGCCCA	
*CmoCh11G004480*	F:TTGGGCCTGAACCTGATGAT	144
	R:CAAGAATAACGAGCCGAGCC	
*CmoCh17G013330*	F:CAATGGCTACGGTTCATGGG	142
	R:TTGGGCAGGAAGGGATCAAT	
*Actin* (*CmoCh04G027660*)	F:AAAGGCAGAGTATGATGAGTCTGG	236
	R:GTCTCGGCAACCAACTCCA	

### Statistical analysis

All data were recorded and compiled using Microsoft Excel 2010. Statistical analyses were performed using SPSS (version 25.0, IBM, Armonk, NY, USA). Data are presented as mean ± standard deviation (SD) of three biological replicates. Significant differences among the lines were determined by one-way analysis of variance (ANOVA), followed by Duncan's multiple range test for post-hoc comparisons. Statistical significance was defined as *p* < 0.05. Correlation analyses were conducted using Pearson’s correlation coefficient. Graphs were generated using Origin 2018 and GraphPad Prism 10.

## Results

### Phenotypic characterization of seed coat structure at different developmental stages

**Fig. 1:**
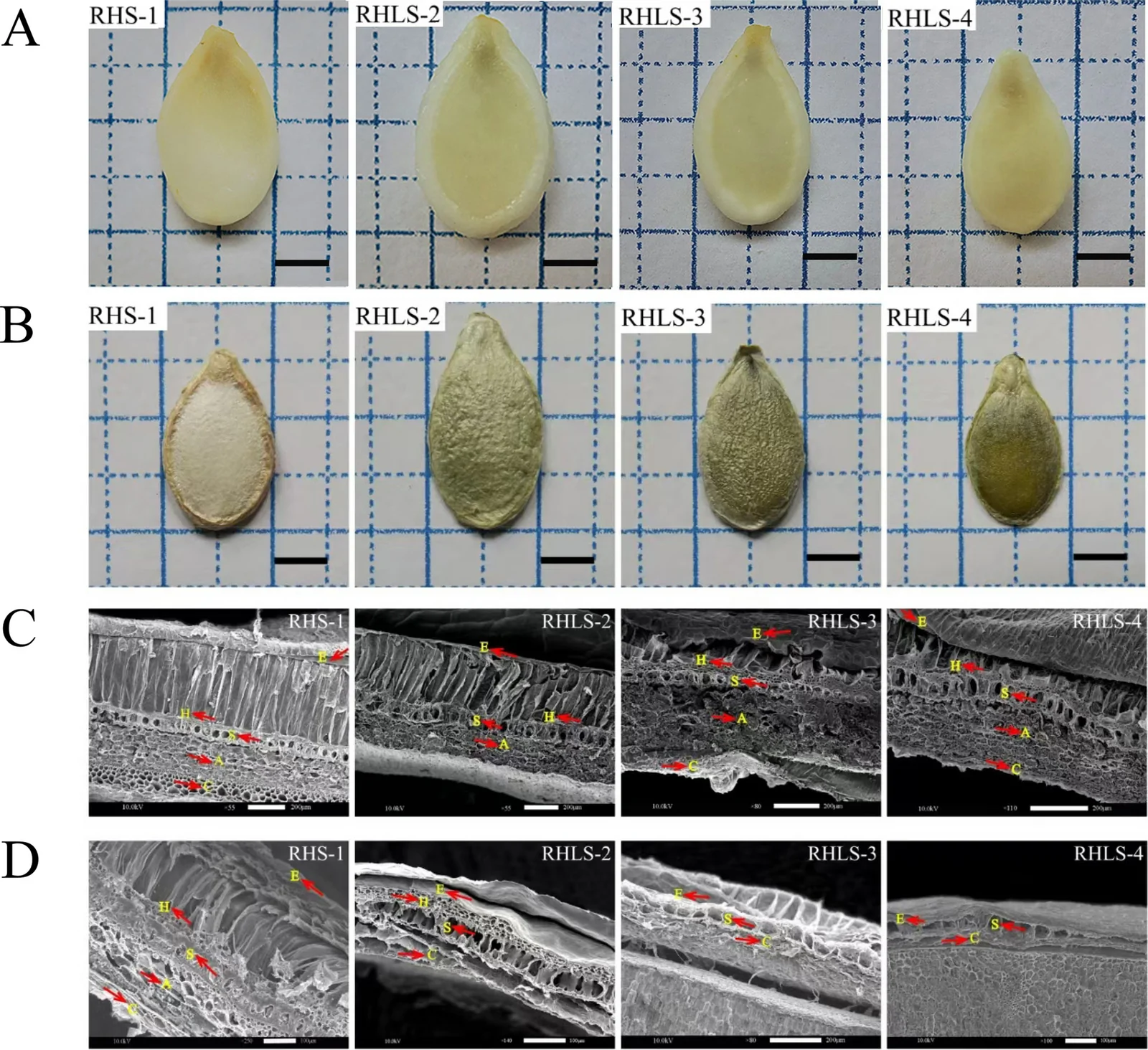
Images of seeds and seed coat structure of hulled and hull-less genotypes. RHS-1, hulled (consumed after dehulling); RHLS-2, RHLS-3, and RHLS-4, hull-less (consumed directly without dehulling), showing moderate, extensive, and nearly complete seed coat degradation phenotype, respectively. **A** Fresh seeds at 20 DPA. Scale bars, 50 mm. **B** Dried seeds at 50 DPA. Scale bars, 50 mm. **C** SEM images of seed coat structure at 20 DPA . Scale bars, 200 μm. **D** SEM images of seed coat structure at 50 DPA. Scale bars: 100 μm for RHS-1, RHLS-2, and RHLS-4; 200 μm for RHLS-3. A, aerenchyma; C, chlorenchyma; E, epidermis; H, hypodermis; S, sclerenchyma

The four seed lines with different genetic backgrounds used in this study exhibited varying seed coat colors and surface morphologies. At 20 DPA, no visible differences in the seed coat were observed between the hulled and hull-less lines at the whole-seed level (Fig. 1A). In contrast, clear phenotypic divergence was evident at 50 DPA (Fig. 1B). At 50 DPA, the seed coat of the hulled wild type (RHS-1) exhibited characteristic beige and highly lignified appearance, with the seed margin showing a yellow–brown coloration (Fig. 1B). The seed coat of the hull-less mutant (RHLS-2) was gray-green, obscuring the seed kernel from visual inspection. The seed coat of the mid-type hull-less mutant (RHLS-3) displayed a gray-green coloration with discontinuous and irregular wavy protuberances on the surface, making the kernel barely perceptible. Notably, no wave protrusions were present in the seed margin or near apical proximal region of the seed, preventing the observation of the seed kernel. The seed coat of the hull-less mutant (RHLS-4) was dark green, while the gray green seed coat remained partially intact at the seed margin and near the top 1/3 of the seed. These observed differences in seed coat colors are likely indicative of the distinct seed coat structures among the four lines.

Further, scanning electron microscopy (SEM) was employed to examine seed coat structure at 20 and 50 DPA of each line to explore the anatomical alterations in the seed coat of different hull-less genotypes at two developmental stages (20 and 50 DPA) relative to hulled wild-type. At 20 DPA, the seed coat of hulled RHS-1 consisted of five distinct layers, organized from the outermost to the innermost: epidermis (E), hypodermis (H), sclerenchyma (S), aerenchyma (A), and chlorenchyma (C). At this stage, all three hull-less lines also exhibited a typical five-layer seed coat structure similar to that of the hulled RHS-1. While, the overall seed coat thickness and the thickness of each individual layers was notably reduced compared in the three hull-less lines compared to the hulled wild type (Fig. 1C, Table 2).

**Table 2:** The thickness of seed coats and five layers of different *C. moschata* lines at 20 DPA and 50 DPA

Line	Days post anthesis (DPA)	Seed coat (μm)	Epidermis layer (μm)	Hypodermis layer (μm)	Sclerenchyma layer (μm)	Aerenchyma layer (μm)	Chlorenchyma layer (μm)
RHS-1	20	816.96 ± 21.03^a^	4.54 ± 1.07^b^	447.34 ± 5.10^a^	54.73 ± 3.37^a^	216.83 ± 24.55^c^	121.26 ± 14.91^a^
	50	391.34 ± 14.28^e^	4.19 ± 1.23^bc^	217.06 ± 6.51^c^	49.46 ± 1.02^ab^	98.15 ± 2.51^e^	19.06 ± 3.11^c^
RHLS-2	20	651.46 ± 21.50^b^	7.15 ± 1.54^a^	370.54 ± 34.69^b^	50.35 ± 3.93^ab^	171.49 ± 8.47^d^	10.62 ± 1.98^ cd^
	50	176.95 ± 38.35^f^	2.75 ± 1.28^c^	37.09 ± 9.78^e^	47.77 ± 3.48^b^	–	–
RHLS-3	20	602.19 ± 3.85^c^	7.42 ± 0.61^a^	168.10 ± 6.33^d^	45.03 ± 3.98^b^	302.16 ± 4.25^a^	4.09 ± 1.23^d^
	50	145.79 ± 6.57^f^	4.39 ± 0.57^bc^	22.11 ± 5.98^e^	36.97 ± 4.69^c^	–	–
RHLS-4	20	542.45 ± 13.91^d^	4.38 ± 0.94^bc^	237.04 ± 6.43^c^	37.31 ± 1.78^c^	264.16 ± 7.57^b^	38.16 ± 6.11^b^
	50	46.65 ± 0.97^ g^	3.04 ± 0.58^bc^	–	10.74 ± 0.69^d^	–	–

At 50 DPA, SEM analysis revealed distinct variations in the seed coat structure among the four tested genotypes (Fig. 1D). The seed coat of RHS-1 still consisted of five distinct layers, with the H and S layers exhibiting high lignification, whereas the A and C layers had collapsed. In RHLS-2, the upright palisade cells in the H layer had collapsed, resulting in the H layer consisting of only three to five layers of round cells, lacking branched interconnections and lignified thickening, the single S layer consisted of thick cells without contents, while the A and C layers had collapsed. In RHLS-3, the H, A, and C layers had collapsed, and none of the remaining seed coat layers showed distinct lignification. In RHLS-4, the S layer, consisting of a single layer of hollow cells, was preserved in very few areas, while the H and A layers had collapsed, and the C layer had undergone compression. At this stage, the substantial structural changes observed in hull-less lines compared with hulled wild type included the complete or partial collapse of the H, A and C layers.

Compared with 20 DPA, the seed coat thickness in RHS-1 at 50 DPA was reduced by 52.10% (Table 2), correspondingly, the thickness of E, H, S, A and C layers in RHS-1 was reduced by 7.71%, 51.48%, 9.63%, 54.73%, and 84.28%, respectively. In RHS-1, the reduction in seed coat thickness at 50 DPA was accompanied by marked decreases in the thickness of the H, A, and C layers. In comparison, the thickness of the H, A and C layers in RHLS-2, RHLS-3 and RHLS-4 at 50 DPA was more significantly reduced, experiencing either complete or partial collapse or severe compression. Based on these quantitative layer thickness measurements, the reduction in seed coat thickness in the mutant lines at 50 DPA was consistently accompanied by the collapse or compression of the H, A and C layers. This result implies that RHLS-2, RHLS-3 and RHLS-4 mutant lines differ from RHS-1 in both the whole-seed phenotype and the seed coat thickness, which is likely associated with different cell wall biosynthesis activities between hulled and hull-less genotypes.

RHS-1, hulled genotype; RHLS-2, RHLS-3, and RHLS-4, hull-less genotypes. Data are presented as mean ± standard error (SE) of three biological replicates. Values with different letter(s) within the same column indicate statistical significance at *p* < 0.05, analyzed by Duncan’s post-hoc test. –, represent collapsed layers where measurements could not be obtained.

### Starch content in the seed coat of different lines at 20 DPA and 50 DPA

**Fig. 2:**
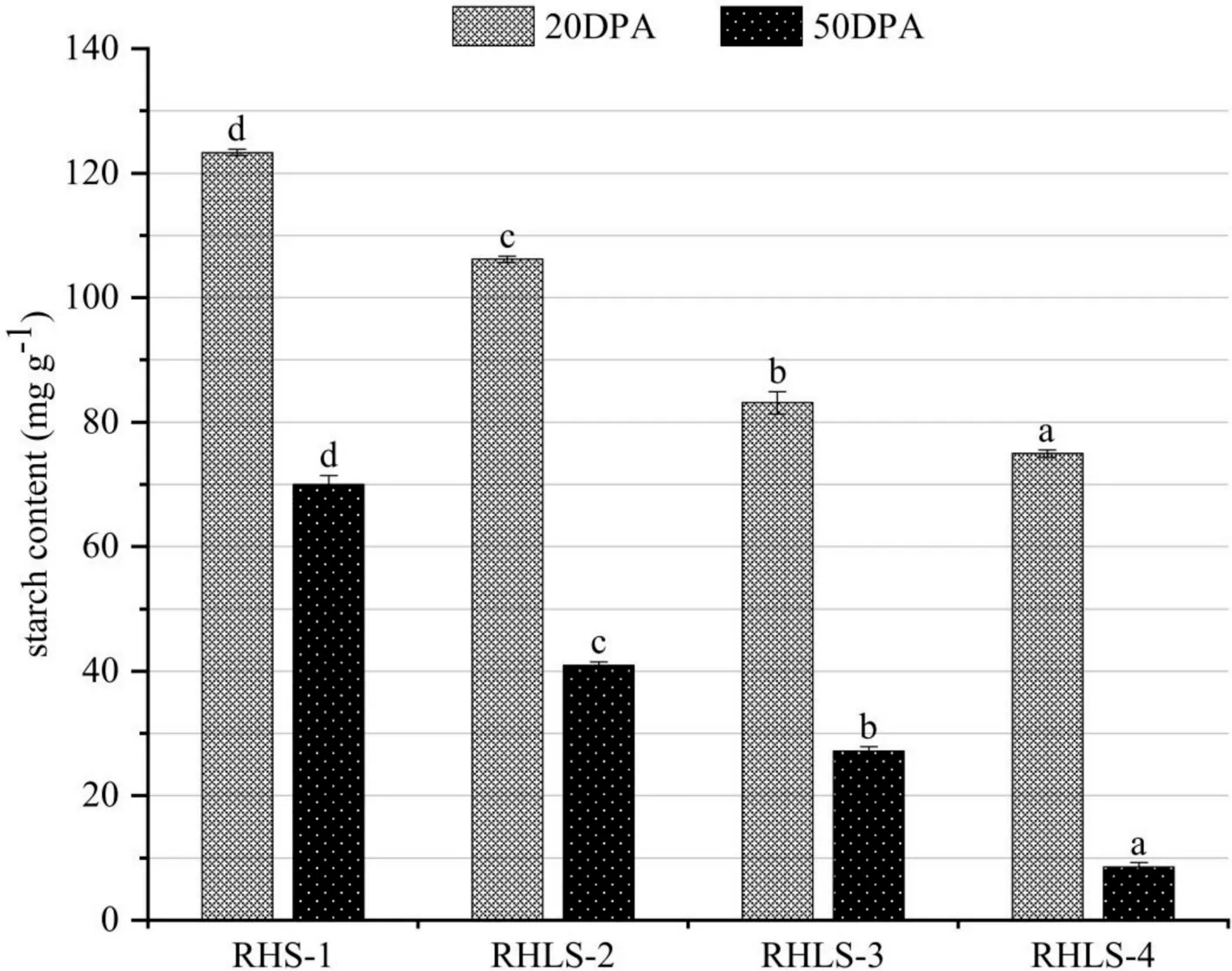
Starch content in seed coat of different genotypes at 20 DPA and 50 DPA. RHS-1, hulled (consumed after dehulling); RHLS-2, RHLS-3, and RHLS-4, hull-less (consumed directly without dehulling), showing moderate, extensive, and nearly complete seed coat degradation phenotype, respectively. Error bars denote mean ± Standard error of three replicates. Bar with different small letter(s) denote significant difference among different genotypes at *p* < 0.05. DPA, days post anthesis

To investigate the relationship between seed coat development and its chemical composition in *C. moschata*, we quantified the starch content in the seed coat of all four lines at both 20 and 50 DPA. As the seed coat development from 20 to 50 DPA, the starch content in the seed coat of all four lines of *C. moschata* decreased significantly (Fig. 2). Specifically, the starch content in the seed coat of RHS-1, RHLS-2, RHLS-3, and RHLS-4 at 50 DPA decreased to 56.78%, 38.55%, 32.66%, and 11.36% of the 20 DPA level, respectively. The seed coats of RHS-1 and RHLS-4 exhibited the lowest and the highest reduction rates of the starch content, respectively. Notably, at both 20 DPA and 50 DPA, the seed coats of RHS-1 and RHLS-4 had the highest and the lowest starch content, respectively. This result implies the potential participation of starch in the development of seed coats; hulled seeds have higher starch content in their seed coats than hull-less seeds. The notably accelerated reduction rates of starch content in the seed coats of hull-less mutant lines probably also contribute to their significantly thinner seed coat phenotype when compared with hulled lines.

### Cell wall constituents in the seed coat of different lines at 20 DPA and 50 DPA

**Fig. 3:**
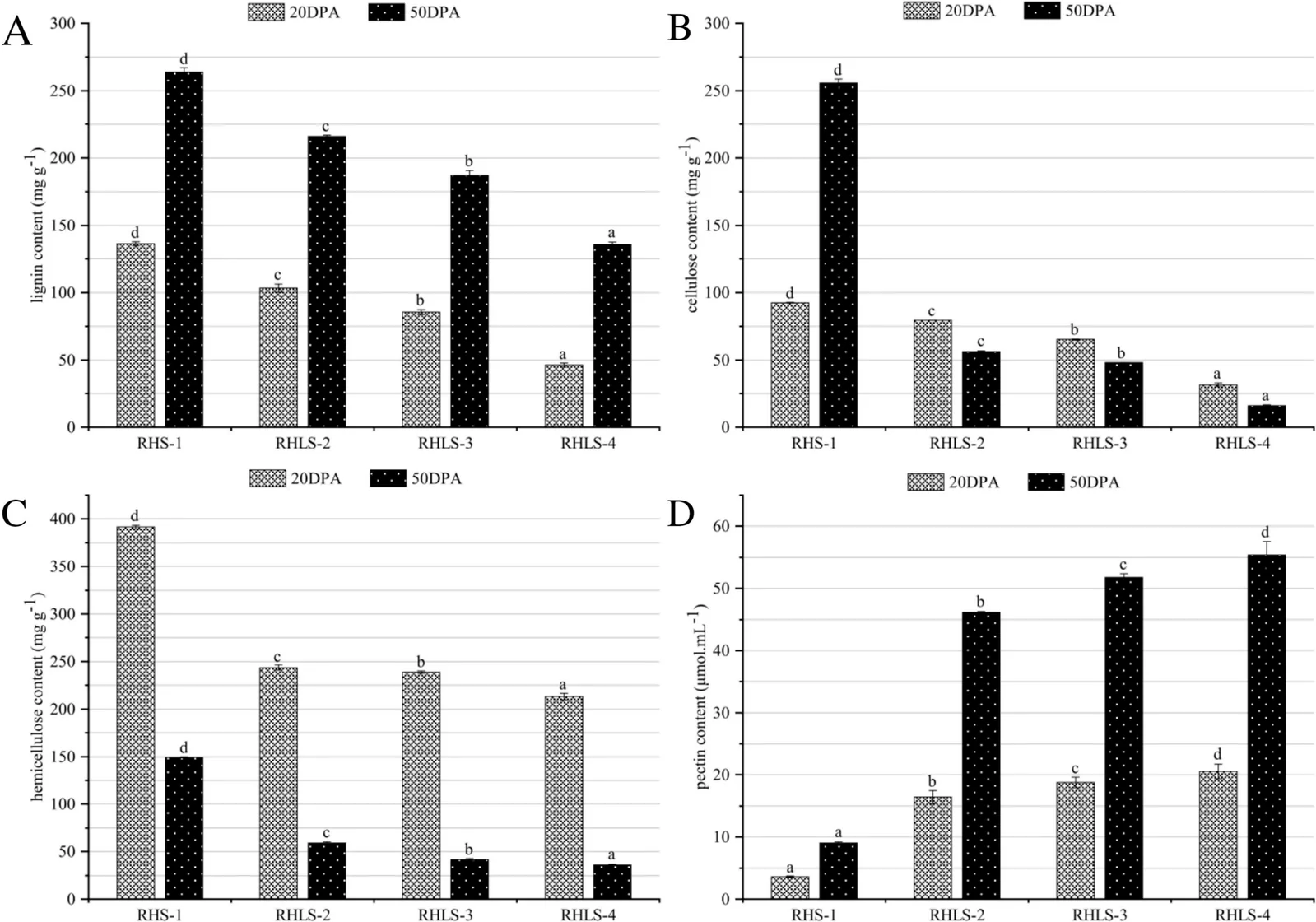
Nonstructural constituents in seed coat of different genotypes at 20 DPA and 50 DPA. **A** Lignin content, (**B**) cellulose content, (**C**) hemicellulose content, and (**D**) pectin content. RHS-1, hulled (consumed after dehulling); RHLS-2, RHLS-3, and RHLS-4, hull-less (consumed directly without dehulling), showing moderate, extensive, and nearly complete seed coat degradation phenotype, respectively. Error bars denote mean ± Standard error of three replicates. Bar with different small letter(s) denote significant difference in genotypes at *p* < 0.05. DPA, days post-anthesis

We further measured the cell wall constituents. Compared with seed coats at 20 DPA, the lignin content in the seed coat of all four tested lines at 50 DPA increased markedly (Fig. 3A). Specifically, the lignin content in the seed coat of RHS-1, RHLS-2, RHLS-3, and RHLS-4 at 50 DPA was 1.94-, 2.09-, 2.19-, and 2.94- fold of that at 20 DPA, respectively. Notably, the seed coat of RHLS-4 had the lowest lignin contents at both 20 DPA and 50 DPA, followed by RHLS-3 and RHLS-2, while the seed coat of RHS-1 had the highest lignin content at both stages. This result suggests that the seed coat of hulled seeds contains a relative higher lignin content compared to those of hull-less seeds.

During the same developmental period, the cellulose content in the seed coat of hull-less seeds decreased significantly, whereas it increased markedly in hulled seeds (Fig. 3B). Specifically, the cellulose content in the seed coat of RHLS-2, RHLS-3, and RHLS-4 at 50 DPA decreased to 0.71-, 0.73-, and 0.51-fold of the 20 DPA level, respectively, while the cellulose content in the seed coat of RHS-1 at 50 DPA was 2.76- fold of that at 20 DPA. At both 20 DPA and 50 DPA, the seed coat of RHLS-4 had the lowest cellulose content, followed by RHLS-3 and RHLS-2, while the seed coat of RHS-1 had the highest cellulose content. This result suggests the potential correlation between the seed coat thinning and reduced cellulose biosynthesis in hull-less lines.

The hemicellulose content in the seed coat of all four tested lines decreased significantly during seed coat development from 20 to 50 DPA (Fig. 3C). Specifically, the hemicellulose content in the seed coat of RHS-1, RHLS-2, RHLS-3 and RHLS-4 at 50 DPA was 38.09%, 24.26%, 17.45%, and 16.93% of those at 20 DPA, respectively. At both 20 DPA and 50 DPA, the seed coats of RHS-1 and RHLS-4 had the highest and the lowest hemicellulose content, respectively. This finding suggests a suppression of hemicellulose biosynthesis in hull-less lines.

The pectin content in the seed coat of all four tested lines increased significantly during seed coat development from 20 to 50 DPA (Fig. 3D). Specifically, the pectin content in the seed coat of RHS-1, RHLS-2, RHLS-3, and RHLS-4 at 50 DPA was 2.51-, 2.81-, 2.75-, and 2.69- fold of that at 20 DPA, respectively. At both 20 DPA and 50 DPA, the seed coats of RHS-1 and RHLS-4 had the lowest and the highest pectin content, respectively. This result suggests an activation of pectin biosynthesis in hull-less lines.

Additional correlation analysis revealed a significant positive correlation between seed coat thickness and lignin content, cellulose content, hemicellulose content, starch content, and sclerenchyma layer thickness at both 20 DPA and 50 DPA. Meanwhile, a significant negative correlation was observed between pectin content and seed coat thickness, lignin content, cellulose content, hemicellulose content, starch content, and sclerenchyma layer thickness at both 20 DPA and 50 DPA (Tables 3 and Table 4). This finding supports the positive involvement of cellulose, semi-cellulose, and lignin, the main primary and secondary cell wall constituents, in the seed coat development in *C. moschata*, and suggests an opposite role of pectin, the main constituent of the middle lamella, in this process.

**Table 3:**
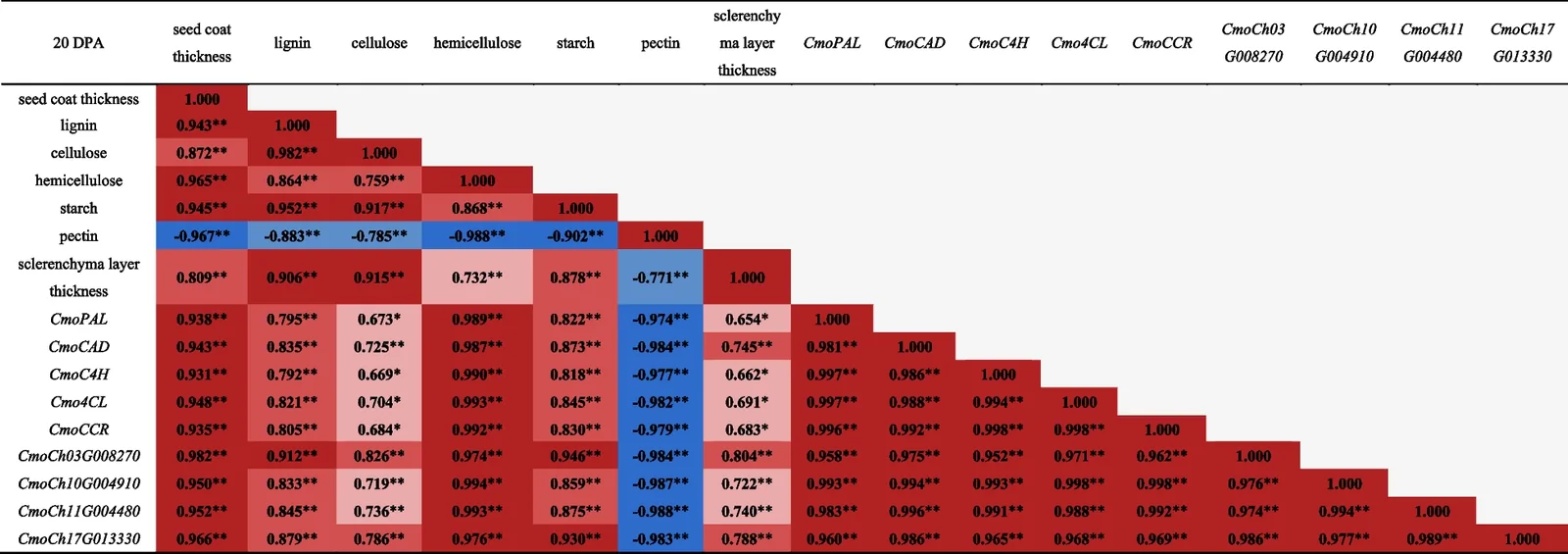
The correlation between different seed coat components and related gene expression levels at 20 DPA

Data are presented as mean ± SD (*n* = 3). **Correlation is significant at the 0.01 level. * Correlation is significant at the 0.05 level. DPA, days post anthesis; *CmoPAL/CmoCh07G009550*, phenylalanine ammonia-lyase; *CmoCAD/CmoCh01G020440*), cinnamyl alcohol dehydrogenase; *CmoC4H*/*CmoCh02G006920*, cinnamate 4-hydroxylase; *Cmo4CL*/*CmoCh01G005470*), 4-coumarate: CoA ligase; *CmoCCR*/*CmoCh17G008650*, cinnamoyl-CoA reductase

**Table 4:**
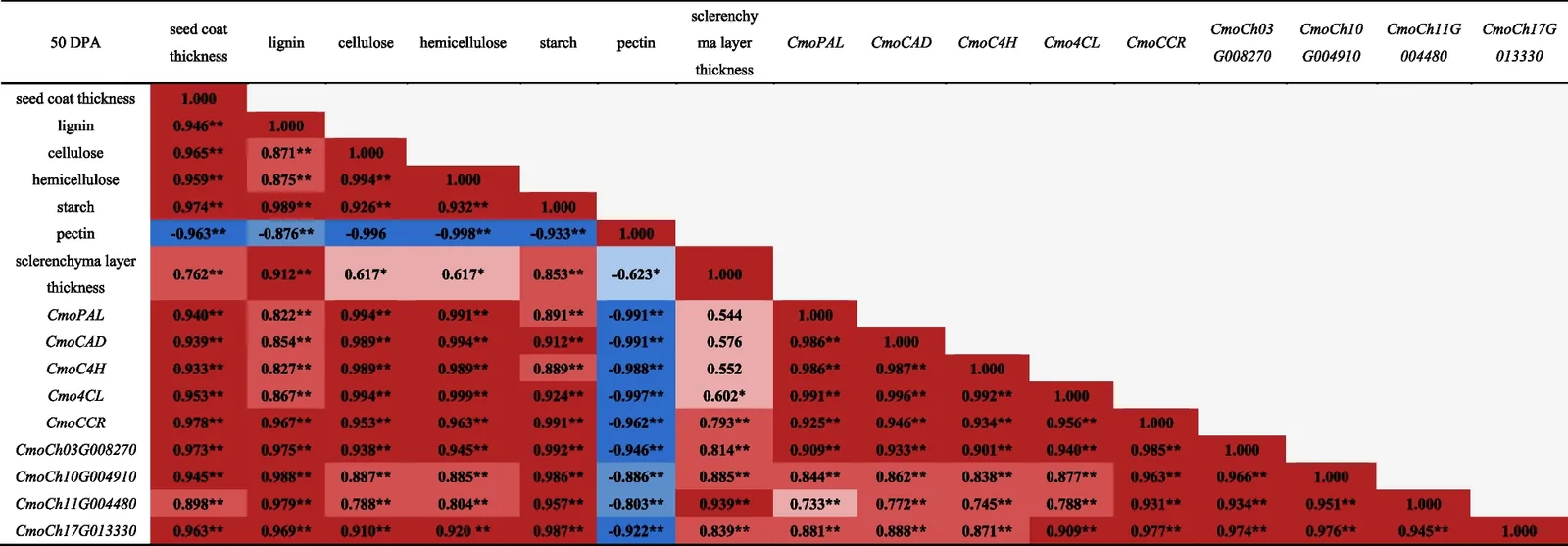
The correlation between different seed coat components and related gene expression levels at 50 DPA

Data are presented as mean ± SD (*n* = 3). **Correlation is significant at the 0.01 level. * Correlation is significant at the 0.05 level. DPA, days post anthesis; *CmoPAL/CmoCh07G009550*, phenylalanine ammonia-lyase; *CmoCAD/CmoCh01G020440*), cinnamyl alcohol dehydrogenase; *CmoC4H*/*CmoCh02G006920*, cinnamate 4-hydroxylase; *Cmo4CL*/*CmoCh01G005470*), 4-coumarate: CoA ligase; *CmoCCR*/*CmoCh17G008650*, cinnamoyl-CoA reductase

### Expression of genes associated with lignin and cellulose biosynthesis in the seed coat of different lines at 20 and 50 DPA

**Fig. 4:**
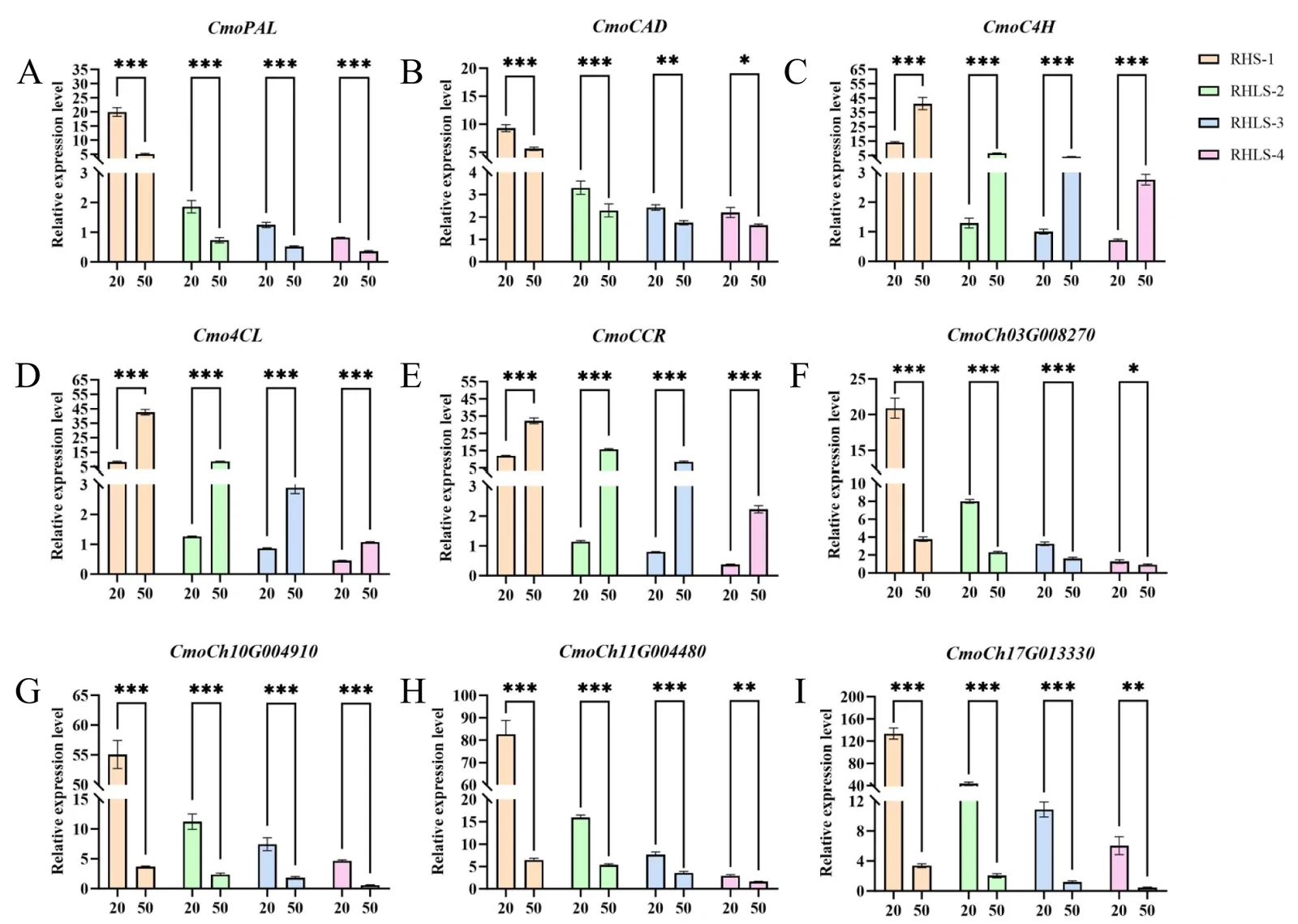
Expression analysis of genes associated with lignin and cellulose metabolism in the seed coat at 20 DPA and 50 DPA of hulled and hull-less genotypes of *C. moschata* using qRT-PCR. **A**  *CmoPAL*/*CmoCh07G009550* (phenylalanine ammonia-lyase), (**B**) *CmoCAD*/*CmoCh01G020440* (cinnamyl alcohol dehydrogenase), (**C**) *CmoC4H*/*CmoCh02G006920* (cinnamate 4-hydroxyla), (**D**) *Cmo4CL*/*CmoCh01G005470* (4-coumarate: CoA ligase), (**E**) *CmoCCR*/*CmoCh17G008650* (cinnamoyl-CoA reductase), (**F**) *CmoCh03G008270*, (**G**) *CmoCh10G004910*, (**H**) *CmoCh11G004480*, and (**I**) *CmoCh17G013330*. RHS-1, hulled (consumed after dehulling); RHLS-2, RHLS-3, and RHLS-4, hull-less (consumed directly without dehulling), showing moderate, extensive, and nearly complete seed coat degradation phenotype, respectively. Actin was used as a normalizer control. Error bars indicate the SE of three biological replicates. *p < 0.05, **p < 0.01, ***p < 0.001

To investigate the molecular basis underlying the observed changes in cell wall components, we examined the expression patterns of nine genes involved in lignin or cellulose biosynthesis using qRT-PCR. The targeted genes comprised five lignin biosynthetic genes (*CmoPAL/CmoCh07G009550*), *CmoCAD/CmoCh01G020440*, *CmoC4H/CmoCh02G006920*), *Cmo4CL/CmoCh01G005470*, and *CmoCCR/CmoCh17G008650*), along with four cellulose biosynthetic genes (*CmoCh03G008270, CmoCh10G004910, CmoCh11G004480,* and *CmoCh17G013330*) (Table 1). The results revealed that the expression levels of the five lignin biosynthetic genes and the four cellulose biosynthetic genes in the seed coat of hulled line are significantly higher than those in hull-less lines at both 20 DPA and 50 DPA (Fig. 4). As seed coat development progressed from 20 to 50 DPA, the expression levels of *CmoPAL*, *CmoCAD*, *CmoCh03G008270, CmoCh10G004910, CmoCh11G004480,* and *CmoCh17G013330* declined significantly, while the expression levels of *CmoC4H*, *Cmo4CL*, and *CmoCCR* increased significantly across all tested lines. Notably, compared to the hulled RHS-1, the expression levels of all five lignin biosynthetic genes and four cellulose biosynthetic genes were decreased, coinciding with the reduction of the seed coat thickness in hull-less lines. This result confirms the positive involvement of lignin biosynthesis and cellulose biosynthesis in seed coat development and their contributions to observed seed coat thinning in hull-less lines.

Correlation analysis results revealed a significant positive correlation between the expression levels of five lignin biosynthetic genes and four cellulose biosynthetic genes with seed coat thickness, lignin content, cellulose content, hemicellulose content, starch content, and sclerenchyma layer thickness at both 20 DPA and 50 DPA. Meanwhile, there was a significant negative correlation between the expression levels of these nine genes and pectin content (Tables 3 and Table 4). This result further supports the distinct roles of different cell wall constituents in the seed coat development in hulled line and seed coat thinning in hull-less mutant lines.

## Discussion

The dehulling procedure in pumpkin seed production has posed challenges to the advancement of the seed industry. In the period between 1870 and 1880, a hull-less mutant was identified within the wild type *C. pepo* [12, 13, 17, 34]*.* subsequent studies have elucidated the anatomical [12–14], physiological [18], biochemical [19], genetic [6, 21–23], and molecular [20] characteristics of this mutant’s seed coat development, contrasting it with that of the wild type (hulled genotype). These findings have provided a robust foundation for breeding initiatives aimed at developing novel varieties with hull-less seeds through the integration of both traditional and modern agricultural biotechnological approaches to meet the demand of the *C. pepo* seed and oil industries.

The discovery of hull-less mutants in the widely cultivated Chinese cultivar, *C. moschata* [7, 8], has provided attractive genetic resources for the development of new pumpkin varieties characterized by high-yield and oil-rich seeds. Owing to their superior taste compared to *C. pepo*, consumers exhibit a preference for seeds from *C. moschata,* particularly those derived from hull-less genotypes [34], which further enhances the desirability of these mutant seeds. Nevertheless, the limited and fragmented knowledge concerning seed coat development in *C. moschata*, relative to that in *C. pepo,* has significantly hindered advancements in leveraging these mutants for *C. moschata* improvement*.*

In this study, we capitalized on the unique characteristics of three hull-less mutants (RILs) with varying degrees of seed coat thickness identified in our lab to conduct a comparative investigation into their anatomical, compositional, and transcriptomic features relative to their hulled wild type line. This investigation elucidated both shared and distinctive properties of seed coat development in *C. moschata* compared to *C. pepo.*

Firstly, the seed coat thickness in seeds of *C. moschata* appears to correlate with seed surface smoothness and seed color (Fig. 1B and Table 2). Specifically, a thinner seed coat is associated with a smooth seed surface and a green color of seed. This correlation facilitates the selection of varieties with desirable hull-less seeds more practical and feasible, making it a viable strategy for future breeding programs, as observed in *C. pepo* [22]. This phenotypic correlation is likely attributable, at least in part, to the collapse of various seed coat layers in hull-less mutants, which may reduce light scattering and thereby enhance the visibility of the underlying green chlorenchyma. In general, the greater the degree of layer collapse, the more pronounced the green coloration of the seeds, consistent with a progressive reduction in seed coat opacity. For instance, as illustrated in Fig. 1 A and Table 2, the seed coat of RHLS-4 was the thinnest, resulting in the smoothest seed surface and the greenest seed color. These findings are consistent with previous studies in hull-less mutant in *C. pepo*, where the collapse of the E, H, S and A seed coat layers leads to an extremely thin, translucent hull that exposes the underlying green colored C layer [12–14]. Similar correlations between seed coat thickness and visible seed traits have been reported in other species. In *Brassica napus*, high-lignin lines exhibit significantly thicker seed coats compared to low-lignin lines [35, 36]. These observations suggest that seed coat thickness, lignin content, and visible seed traits are tightly linked through conserved developmental programs across diverse species.

Secondly, from an anatomical perspective, the reduction in seed coat thickness observed in both hulled wild type and hull-less mutants were morphologically associated with the diminution or collapse of the H, A, and C layers (Fig. 1D and Table 2). However, the extent of this reduction varied markedly between hulled and hull-less genotypes: in the hulled wild type, these layers decreased moderately during development, whereas in hull-less mutants, they underwent pronounced collapse or, in some cases, complete disappearance at 50 DPA. Notably, this layer reduction was progressively more severe from RHLS-2 to RHLS-4, correlating with their increasing degrees of seed coat thinning. The hulled wild type RHS-1 in *C. moschata* exhibited a characteristic five-layer seed coat structure at 50 DPA as that in *C. pepo* [12, 13]. In line with our earlier findings [7], the S and H layers of RHS-1 exhibited significantly enhanced lignification (Fig. 1C). Various hull-less mutants displayed varying degrees of seed coat layer collapse. For instance, the seed coat of RHLS-4 at 50 DPA consisted primarily of the single E layer cell, the large, hollow S layer cells and the underlying C layer cells, forming a papery thin transparent hull that revealed the green color of the chlorenchyma layer, which is rich in protochlorophyll pigment [14]. Other hull-less mutants, such as RHLS-2 and RHLS-3, exhibited varying degrees of collapse or compression in the S, H, and A layers, suggesting intermediate lines between RHS-1 and RHLS-4. Notably, the E layer in the seed coat of hull-less mutants remained comparable to that of the hulled wild type in *C. moschata*, without undergoing the significant collapse observed in *C. pepo* [12–14]. Nevertheless, the reduction in seed coat thickness is primarily attributable to the collapse and compression of the H, A, and C layers, rather than a uniform decrease across all seed coat layers. In contrast, the S layer, the main lignified structural layer, remained largely intact and continued to accumulate lignin and cellulose during development. This explains the concurrent increase in these components despite overall seed coat thinning. However, these findings are based only on morphological observations, so we cannot conclude the underlying mechanisms. This pattern is consistent with previous reports in *C. pepo* [12–14] and *Arabidopsis* [27, 28], where lignification occurs in specific layers while others undergo degeneration. The anatomical basis of seed coat thinning has also been investigated in other plant systems. In *Arabidopsis thaliana*, the seed coat consists of five distinct layers, with the inner integument undergoing lignification during development, and transcription factors such as *NST1* and *MYB46* have been identified as master regulators of secondary cell wall formation [27, 28]. In *B. napus*, the palisade layer is a primary contributor to seed coat thickness differences between high- and low- lignin lines [32]. These studies, together with our findings in *Cucurbita*, suggest that layer-specific lignification and collapse may represent general correlates of seed coat morphological variation across angiosperms.

Thirdly, studies in *C. pepo* proposed that cell wall components, such as cellulose, hemicellulose, lignin and pectin, are major contributors to seed coat thickening, and that these structural components, rather than non-structural components such as amino acids, starch, and lipids, may underlie the reduction in seed coat thickness in hull-less mutant lines [6, 18, 23]. Particularly, lignin biosynthesis plays an important role in the seed coat development and its reduction results in the hull-less phenotype in *C. pepo* [18–20]. Our findings in this study confirmed significant compositional changes in both cell wall constitutes and non-cell wall components in seed coat of both hulled and hull-less lines of *C. moschata* during seed coat development. Specifically, the seed coat of hull-less mutants exhibited lower levels of lignin, cellulose and hemicellulose compared to those of hulled wild type (Figs. 2 and 3), consistent with observations in *C. pepo*. Different from *C. pepo*, the seed coats of hull-less mutants in *C. moschata* contained higher levels of pectin than those of hulled wild type (Fig. 3D), as supported by correlation analysis (Table 3 and Table 4). In addition, hull-less mutant seed coat had lower starch content than those of hulled wild type (Fig. 2), implying the potential involvement of starch metabolism in seed coat reduction in hull-less mutants of *C. moschata*. Considering the intricate relationship between cell wall components and non-structural metabolites such as starch [36], future studies should focus in the allocation of carbon to starch or cellulose in the seed coat of hull-less mutants of *C. moschata* during seed coat development. These results suggest that the metabolic homeostasis among non-structural and structural constitutes in the seed coat of hull-less mutants is disrupted, with reduced accumulation of starch, lignin, cellulose and hemicellulose but increased accumulation of pectin in hull-less mutants compared with the hulled wild type. The relationship between lignin and seed coat thickness is conserved across species. In *B. napus*, high-lignin lines exhibit significantly thicker seed coats than low-lignin lines, with genes encoding lignin biosynthetic enzymes (BnPAL, BnC4H, Bn4CL) highly expressed in high-lignin lines [28, 29]. In *Arabidopsis*, *NST1* and *MYB46* function as master regulators of secondary wall formation by activating downstream lignin biosynthetic genes, with their activity regulated at both transcriptional and post-translational levels [24, 25]. These cross-species comparisons indicate that lignin accumulation is a conserved determinant of seed coat thickness and structural properties. Nevertheless, lignin is often deposited in the secondary wall to increase the hardness of the cell wall. In this study, lignified H layer and S layer showed different anatomic changes during seed coat development, indicating that the specifical role of the lignified H layer in the seed coat thinning in *C. moschata*.

Among all the tested structural and non-structural components, the trends in the content changes of starch, lignin, hemicellulose, and pectin during seed coat development from 20 to 50 DPA were similar in hulled and hull-less genotypes, albeit with varying degrees of variation. However, the trend in cellulose content differed markedly; it consistently increased in hulled wild type but decreased in hull-less mutants from 20 to 50 DPA (Figs. 2 and 3). This result raises the possibility that cellulose may be involved in the reduction of seed coat thickness in hull-less mutant lines of *C. moschata*. Consistently, the reduction in hemicellulose content during seed coat development from 20 to 50 DPA in hull-less RHLS-4 was significantly greater than that in hulled RHS-1 (Fig. 3C). In addition, the reduction in starch content during the same period in RHLS-4 was also significantly greater than that in RHS-1 (Fig. 2). Furthermore, the correlation coefficient between cellulose or starch and seed coat thickness at 50 DPA was the highest (Table 4). Moreover, during seed coat development from 20 to 50 DPA, the proportion of cellulose decreased dramatically in hull-less mutants but increased significantly in hulled wild type. These correlative findings suggest that cellulose may be more closely associated with seed coat thinning than lignin in hull-less mutants of *C. moschata*. When integrated with the anatomical observations of H, A, and C layer collapse, these biochemical correlations point to a multi-faceted process in which cell wall compositional changes and physical layer collapse act in concert to reduce seed coat thickness. Notably, the divergent cellulose trends between hulled and hull-less lines during development suggest that cellulose metabolism, rather than lignin alone, may be a critical determinant of the hull-less phenotype in *C. moschata,* a feature that distinguishes it from the lignin-centric model proposed for *C. pepo*. However, qRT-PCR did not support this idea (Fig. 4F-I), indicating the existence of additional regulatory mechanisms (for example post-transcriptional regulation) underlying the seed coat thinning in *C. moschata*; further functional studies are required to establish such a causal relationship.

The finding of higher pectin content in the seed coat of hull-less mutants than that of hulled wild type in *C. moschata* (Fig. 3D) contradicted the finding from a previous study in *C. pepo*, where the pectin content in the seed coat of hull-less mutants was less than that of hulled wild type [6]. While we cannot yet assert that this phenomenon is specific to *C. moschata*, the increased accumulation of pectin in the seed coat of hull-less mutant holds significant industrial value. This is due to the widespread use of pectin in the food processing industries, attributed to its gelling properties, which serve various functions, including as a stabilizer in jams and jellies, an edible film, and a thickening agent [37].The pectin content in the seed coat of hull-less RHLS-4 was more than sixfold that of hulled RHS-1 (Fig. 3D), making RHLS-4 particularly appealing to the market. Considering that pectin and hemicellulose fill the spaces between cellulose microfibrils-based cell wall, providing elasticity and adhesiveness, and that pectin is the main composition of the middle lamella, providing adhesion of adjacent cells, further studies need to focus on its functional importance in seed coat thinning in hull-less mutant lines, for example, its role in seed desiccation, an important process of seed maturation.

Fourthly, the collapse of cell layers and change in compositions in hull-less mutants in *C. pepo* are accompanied by a reduced expression of genes involved in lignin biosynthesis [35]. Our studies in *C. moschata* confirmed its conservation in pumpkin (Fig. 4A-E). The higher expression of *CmoPAL*, *CmoCAD*, *CmoC4H*, *Cmo4CL*, and *CmoCCR* in hulled wild type compared to those in hull-less lines (Fig. 4A-E) was consistent with higher lignin content in hulled wild type than those in hull-less mutant lines (Fig. 3 A). This result supports the notion that lignin biosynthesis contributes to the seed coat development in *C. moschata*. During seed coat development from 20 to 50 DPA, the expression of *CmoC4H*, *Cmo4CL*, and *CmoCCR* was upregulated, while that of *CmoPAL* and *CmoCAD* was downregulated. Given the increase in lignin content during seed coat development, it is likely that *CmoC4H*, *Cmo4CL*, and *CmoCCR* play a more important role in lignin biosynthesis than *CmoPAL* and *CmoCAD* in *C. moschata*. This result differs from the findings reported in *C. pepo* [20], and may explain the increase of lignin in the seed coat of both hulled and hull-less lines at 50 DPA (Fig. 3A). However, this result aligns with observations in other species. In *B. napus*, the expression of *BnPAL*, *BnC4H*, and *Bn4CL* is significantly higher in high-lignin seed coats than those in low-lignin seed coats [29]. In *Arabidopsis*, the master secondary wall formation regulators (*NST1* and *MYB46*) activate downstream lignin biosynthetic genes, with their activity regulated at both transcriptional and post-translational levels [24, 25]. Collectively, these cross-species comparisons support a conserved model in which a NAC-MYB transcriptional cascade governs secondary wall deposition, with lignin as a key output determining seed coat mechanical properties and thickness. Within this framework, the reduced lignin accumulation in hull-less mutants of both *C. pepo* and *C. moschata* likely reflects the disruption of this conserved regulatory module, while the species-specific differences in pectin and cellulose metabolism suggest that downstream metabolic outputs may have diverged during evolution.

The expression patterns of four cellulose biosynthetic genes (*CmoCh03G008270, CmoCh10G004910, CmoCh11G004480,* and *CmoCh17G013330*) exhibited similar trends during seed coat development from 20 to 50 DPA in both hulled and hull-less lines, with lower expression levels observed in hull-less mutants (Fig. 4F-I). These trends were consistent with observed changes in hemicellulose content in the seed coat of all four lines (Fig. 3 C). However, they were not consistent with the cellulose content, particularly in the seed coat of hulled RHS-1 wild type (Fig. 3B). This discrepancy was not reported in *C. pepo* [20, 35]. It is likely that, in addition to transcriptional regulation, other regulatory mechanisms, such as posttranscriptional regulation, play a role in the regulation of cellulose biosynthesis in the seed coat of *C. moschata*, which warrants future investigations. Such post-transcriptional regulation of cellulose biosynthesis has also been suggested in other plant systems. In *Arabidopsis*, the stability of MYB46 protein (a key activator of secondary wall biosynthesis) is negatively regulated by mitogen-activated protein kinase 6 (MPK6) at the post-translational level [25]. Collectively, the gene expression data support the notion that *C. moschata* and *C. pepo* share both conserved and distinct metabolic pathways involved in the seed coat development [31].

Lastly, as reported in both *C. moschata* [8] and *C. pepo* [6, 21, 22], NAC transcription factors play a crucial role in the regulation of seed coat development in both species. It is intriguing to explore why mutations in the same gene lead to distinct anatomical, compositional, and transcriptomic alterations in the mutants, whether additional metabolic pathways are implicated in seed coat development, and how NAC coordinate the regulation of these metabolic pathways. The central role of *NAC* transcription factors in regulating secondary cell wall formation and seed coat development is a conserved theme across plant species. In *Arabidopsis*, *NST1*, *NST2*, and *NST3* function as master switches that directly activate *MYB46* and *MYB83*, which in turn regulate downstream genes involved in cellulose, hemicellulose, and lignin biosynthesis [24]. In *B. napus*, *BnNAC080* and *BnMYB9* are upregulated in high-lignin seed coats [28, 29]. This conservation of the NAC-MYB regulatory module across distantly related angiosperms suggests that the hull-less phenotype in *Cucurbita* results from disruption of a fundamental, evolutionarily conserved program of secondary cell wall deposition in the seed coat. Synthesizing our anatomical, biochemical, and transcriptomic data, we propose a unified framework for seed coat thinning in *C. moschata* hull-less mutants. At the structural level, the progressive collapse of the H, A, and C layers constitutes the primary anatomical basis. At the compositional level, this collapse is accompanied by altered cell wall structural constituents (reduced lignin, cellulose, and hemicellulose, but elevated pectin) and non-cell wall structural constituents (reduced starch), suggesting a global reprogramming of cell wall and non-cell wall metabolisms. At the transcriptional level, the downregulation of lignin and cellulose biosynthetic genes in hull-less mutants is consistent with reduced secondary wall deposition. Notably, the discordance between *CesA* expression and cellulose content, together with the elevated pectin accumulation not observed in *C. pepo*, highlights species-specific regulatory and metabolic divergence. We hypothesize that these differences may arise from altered carbon flux partitioning between structural and non-structural carbohydrate pools, a possibility that warrants future investigation through metabolic flux analysis and functional validation of candidate genes.

We acknowledge that the present gene expression study examined only nine candidate genes and did not perform genome-wide transcriptomic or functional analyses. Future studies employing RNA-seq and gene functional validation (e.g., VIGS or CRISPR/Cas9) will be required to fully decipher the regulatory network controlling seed coat development and thinning in *C. moschata*. Nonetheless, the comparative data presented here provide a valuable basis for future investigations.

## Conclusion

In conclusion, this study presents a comparative analysis of anatomical, compositional, and gene expression differences between hulled (RHS-1) and hull-less (RHLS-2, RHLS-3, and RHLS-4) lines of *C. moschata*. The distinct patterns observed among the four lines reveal a progressive degradation phenotype characterized by the collapse of specific cell layers (H, A, and C) and reduced lignin and cellulose deposition during seed coat development in pumpkins. Changes in cell wall composition and gene expression were correlated with seed coat thickness, suggesting that these factors may be associated with the hull-less phenotype. However, the correlative nature of these data precludes definitive causal conclusions, and the specific roles of NAC transcription factors or carbon allocation in this process remain to be established. While the underlying mechanisms of seed coat thinning in hull-less lines require further investigations, our results provide a comprehensive comparative characterization of seed coat development in *C. moschata* and identify several candidate genes, particularly those involved in lignin and cellulose biosynthesis, for future functional studies. These findings may also provide a valuable foundation for breeding hull-less varieties to meet growing market demand.

## Data Availability

All data supporting this article are available from the corresponding author upon reasonable request.

## References

[1] Bergantin C, Maietti A, Tedeschi P, Font G, Manyes L, Marchetti N. HPLC-UV/Vis-APCI-MS/MS determination of major carotenoids and their bioaccessibility from “Delica” (*Cucurbita maxima*) and “Violina” (*Cucurbita moschata*) pumpkins as food traceability markers. Molecules. 2018;23:2791. 30373266 10.3390/molecules23112791 PMC6278257

[2] Vinayashree S, Vasu P. Biochemical, nutritional and functional properties of protein isolate and fractions from pumpkin (*Cucurbita moschata* var. Kashi Harit) seeds. Food Chem. 2021;340:128177. 33002826 10.1016/j.foodchem.2020.128177

[3] Dash P, Ghosh G. Amino acid profiling and antimicrobial activity of *Cucurbita moschata* and *Lagenaria siceraria* seed protein hydrolysates. Nat Prod Res. 2018;32:2050–3. 28783965 10.1080/14786419.2017.1359174

[4] Kim EY, Chang WK, Chang PS. Purification and characterization of a novel acid-tolerant and heterodimeric β-glucosidase from pumpkin (*Cucurbita moschata*) seed. J Biosci Bioeng. 2021;132:125–31. 34078567 10.1016/j.jbiosc.2021.04.004

[5] Šamec D, Loizzo MR, Gortzi O, Çankaya İT, Tundis R, Suntar İ, et al. The potential of pumpkin seed oil as a functional food-a comprehensive review of chemical composition, health benefits, and safety. Compr Rev Food Sci Food Saf. 2022;21:4422–46. 35904246 10.1111/1541-4337.13013

[6] Kaur B, Garcha KS, Bhatia D, Khosa JS, Sharma M, Mittal A, et al. Identifification of single major QTL and candidate gene(s) governing hull-less seed trait in pumpkin. Front Plant Sci. 2022;13:948106. 36035714 10.3389/fpls.2022.948106 PMC9406289

[7] Shen Q, Wu LY, Zhao XH, Li YL. Comparative analysis of electron microscopic structure of seed testa, lignin and biosynthesis related enzyme activities in hulled and hull-less seeds of *Cucurbita moschata*. Sci Hortic. 2019;245:137–43.

[8] Shen Q, Weng YQ. Alternative splicing of NAC transcription factor gene *CmNST1* is associated with naked seed mutation in pumpkin, *Cucurbita moschata*. Genes. 2023;14:962. 37239322 10.3390/genes14050962 PMC10217548

[9] Ren JZ, Wei ZX. Market changes and industrial development strategies of seed pumpkin. China Veg. 2018;5:16–19 (in Chinese).

[10] Shi YZ, Ma W, Sun TZ, Duan Y, Wang CL. Progress in seed pumpkin breeding. China Veg. 2019;08:13–20 (in Chinese).

[11] Lelley T, Loy B, Murkovic M. Hull-less oil seed pumpkin. Oil. Crops. 2010;4:469–92.

[12] Zraidi A, Pachner M, Lelley T. On the genetics and histology of the hull-less character of styrian lipid-pumpkin (*Cucurbita pepo* L.). Cucurbit Genet Coop Rep. 2003;26:57–61.

[13] Stuart SG, Loy JB. Comparison of testa development in normal and hull-less seeded strains of *Cucurbita pepo* L. Bot Gaz. 1983;144:491–500.

[14] Kreft M, Zorec R, Janeš D, Kreft S. Histolocalisation of the oil and pigments in the pumpkin seed. Ann Appl Biol. 2009;154:413–8.

[15] Murovec J, Drašlar K, Bohanec B. Detailed analysis of *Cucurbita pepo* seed coat types and structures with scanning electron microscopy. Botany. 2012;90:1161–9.

[16] Bezold TN, Loy JB, Minocha SC. Changes in the cellular content of polyamines in different tissues of seed and fruit of a normal and a hull-less seed variety of pumpkin during development. Plant Sci. 2003;164:743–52.

[17] Loy JB. Seed development in *Cucurbita pepo*: an overview with emphasis on hull less seeded genotypes of pumpkin. Cucurbit Genet Coop Rep. 2000;23:89–95.

[18] Arshdeep K, Gagandeep KC, Ajmer SD. Understanding of histological and physiological alterations in hull-less seeded pumpkin. Sci Hor. 2022;304:111321.

[19] Xue YY, Shi GY, Xu BL, Chen RX. Studies on morphology of seed coat development and its related enzyme activity assay in *Cucurbita pepo*. Acta Prataculturae Sin. 2011;20:23–30 (in Chinese, with English abstract).

[20] Zhao XY, Yang J, Qu F, Ma GP, Tian Y, Wen LH. Cloning and expression analysis of key genes for lignin synthesis in *Cucurbita pepo* L. Braz J Bot. 2022;45:909–16.

[21] Meru G, Fu Y, Shrestha S, Michael VNE, Dorval M, Mainviel R. Genomic position and markers associated with the hull-less seed trait in pumpkin. Plants. 2022;11:1238. 35567238 10.3390/plants11091238 PMC9103792

[22] Fabrizio J, LaPlant K, Wyatt L, Inzinna G, Li L, Mazourek M. Fine mapping and identifcation of candidate genes for the hull-less seed phenotype in *Cucurbita pepo*. Euphytica. 2022;218:160.

[23] Lv XL, Shi L, Zhao M, Li Z, Liao N, Meng Y, et al. A natural mutation of the *NST1* gene arrests secondary cell wall biosynthesis in the seed coat of a hull-less pumpkin accession. Hortic Res. 2022;9:136. 10.1093/hr/uhac136 PMC9437724 36072840

[24] Liu C, Yu H, Rao X, Li L, Dixon RA. Abscisic acid regulates secondary cell-wall formation and lignin deposition in *Arabidopsis thaliana* through phosphorylation of *NST1*. Proc Natl Acad Sci U S A. 2021;118(5):e2010911118. 33495344 10.1073/pnas.2010911118 PMC7865148

[25] Im JH, Ko JH, Kim WC, Crain B, Keathley D, Han KH. Mitogen-activated protein kinase 6 negatively regulates secondary wall biosynthesis by modulating MYB46 protein stability in *Arabidopsis thaliana*. PLoS Genet. 2021;17(4):e1009510. 33826618 10.1371/journal.pgen.1009510 PMC8055014

[26] Liu Y, Wu Q, Qin Z, Huang J. Transcription factor *OsNAC055* regulates GA-mediated lignin biosynthesis in rice straw. Plant Sci. 2022;325:111455. 36152809 10.1016/j.plantsci.2022.111455

[27] Jung SE, Kim TH, Shim JS, Bang SW, Bin YH, Oh SH, et al. Rice *NAC17* transcription factor enhances drought tolerance by modulating lignin accumulation. Plant Sci. 2022;323:111404. 35914574 10.1016/j.plantsci.2022.111404

[28] Zhang Y, Zhang H, Zhao H, Xia Y, Zheng X, Fan R, et al. Multi-omics analysis dissects the genetic architecture of seed coat content in *Brassica napus*. Genome Biol. 2022;23(1):86. 35346318 10.1186/s13059-022-02647-5 PMC8962237

[29] Ding Y, Yu S, Wang J, Li M, Qu C, Li J, et al. Comparative transcriptomic analysis of seed coats with high and low lignin contents reveals lignin and flavonoid biosynthesis in *Brassica napus*. BMC Plant Biol. 2021;21(1):246. 34051742 10.1186/s12870-021-03030-5 PMC8164251

[30] Ling YR. A new compendium of materia medica. (Pharmaceutical Botany and China Medicinal Plants). Beijing: Huayu Nature Book Trade Co. Ltd; 1995.

[31] Zhou XL. Study on the breeding and genetic rules of hull-less *Cucurbita moschata*. J Shanxi Agric Sci. 1983;12:11–2 (in Chinese).

[32] Zhou XL. A study on the breeding of hull-less *Cucurbita moschata* and its genetic behavior. Acta Hortic Sin. 1987;14:115–8 (in Chinese).

[33] Livak KJ, Schmittgen TD. Analysis of relative gene expression data using real-time quantitative PCR and the 2^-∆∆CT^ method. Methods Cell Sci. 2001;25:402–8. 10.1006/meth.2001.1262 11846609

[34] Teppner H. *Cururbita pepo* (Cucurbitaceae): history, seed coat types, thin coated seeds and their genetics. Phyton-Int J Exp Bot. 2000;40:1–42.

[35] Bezold TN, Mathews D, Loy JB, Minocha SC. Molecular analysis of the hull-less seed trait in pumpkin:expression profiles of genes related to seed coat development. Seed Sci Res. 2005;3:205–17.

[36] Pottier D, Roitsch T, Persson S. Cell wall regulation by carbon allocation and sugar signaling. Cell Surf. 2023;9:100096. 10.1016/j.tcsw.2023.100096 PMC10311191 37396713

[37] Lalnunthari C, Devi LM, Badwaik LS. Extraction of protein and pectin from pumpkin industry by-products and their utilization for developing edible film. J Food Sci Technol. 2020;57:1807–16. 32327791 10.1007/s13197-019-04214-6 PMC7171003

